# Rapid Discrimination of Gram-Positive and Gram-Negative Bacteria in Liquid Samples by Using NaOH-Sodium Dodecyl Sulfate Solution and Flow Cytometry

**DOI:** 10.1371/journal.pone.0047093

**Published:** 2012-10-15

**Authors:** Atsushi Wada, Mari Kono, Sawako Kawauchi, Yuri Takagi, Takashi Morikawa, Kunihiro Funakoshi

**Affiliations:** Cell Analysis Center, Scientific Affairs, Sysmex Corporation, Nishi-ku, Kobe, Hyogo, Japan; Charité, Campus Benjamin Franklin, Germany

## Abstract

**Background:**

For precise diagnosis of urinary tract infections (UTI), and selection of the appropriate prescriptions for their treatment, we explored a simple and rapid method of discriminating gram-positive and gram-negative bacteria in liquid samples.

**Methodology/Principal Findings:**

We employed the NaOH-sodium dodecyl sulfate (SDS) solution conventionally used for plasmid extraction from *Escherichia coli* and the automated urine particle analyzer UF-1000i (Sysmex Corporation) for our novel method. The NaOH-SDS solution was used to determine differences in the cell wall structures between gram-positive and gram-negative bacteria, since the tolerance to such chemicals reflects the thickness and structural differences of bacterial cell walls. The UF-1000i instrument was used as a quantitative bacterial counter. We found that gram-negative bacteria, including *E. coli*, in liquid culture could easily be lysed by direct addition of equal volumes of NaOH-SDS solution. In contrast, *Enterococcus faecalis*, which is a gram-positive bacterium, could not be completely lysed by the solution. We then optimized the reaction time of the NaOH-SDS treatment at room temperature by using 3 gram-positive and 4 gram-negative bacterial strains and determined that the optimum reaction time was 5 min. Finally, in order to evaluate the generalizability of this method, we treated 8 gram-positive strains and 8 gram-negative strains, or 4 gram-positive and 4 gram-negative strains incubated in voluntary urine from healthy volunteers in the same way and demonstrated that all the gram-positive bacteria were discriminated quantitatively from gram negative bacteria using this method.

**Conclusions/Significance:**

Using our new method, we could easily discriminate gram-positive and gram-negative bacteria in liquid culture media within 10 min. This simple and rapid method may be useful for determining the treatment course of patients with UTIs, especially for those without a prior history of UTIs. The method may be easily applied in order to obtain additional information for clinical prescriptions from bacteriuria.

## Introduction

During the initial treatment of infectious diseases, including urinary tract infections (UTIs), physicians prescribe antibiotics empirically because of a lack of information on the pathogen. Although the burden of UTIs for most otherwise healthy patients is usually not considerable, prompt diagnosis and treatment are important, especially for certain subpopulations, such as children, pregnant women, and the elderly. Delays in the diagnosis and treatment of UTIs increase the risk of severe outcomes, such as tissue invasion and sepsis [1]–[3].

In order to prescribe antibiotics properly at the first visit of patients with infections, information on the infecting organisms is helpful. The definitive diagnosis of a UTI is generally made on the basis of the presence of a pathogen in midstream urine specimens [1]–[3]. Although the gold standard for bacterial detection and enumeration is a semiquantitative urinary culture, the detection and enumeration requires an incubation that is at least overnight, and the identification of the relevant bacteria and susceptibility testing takes an additional 24 to 48 h. For these reasons, information about the pathogen from laboratory cultures is usually not available at the first visit of the patient with a UTI. Moreover, there is a risk of overlooking the real pathogen because not all bacteria that cause UTIs grow with the standard cultivation method [4], [5]. Other methods, dipstick and microscopic analyses, are also frequently used for urinary screening. They are not as time-consuming as clinical cultures. Nitrate reductase assays on dipsticks have been used as a rapid and cheap screening method, but they lack accuracy and precision [6]. Microscopic sediment analyses have been used as screening methods and are more reliable for finding bacteria than dipsticks. Urinary gram stain is a reliable diagnostic test for detection of bacteria in UTIs. Detection of bacteriuria by microscopy with Gram stain is the single best test and, if available locally and reported rapidly, is the only test that would need to be done to guide empirical treatment of patients with antibiotics [7]. However, these microscopic methods are time consuming and dependent on the skill and experience of the technician [8]. In addition, some new methods that detect nucleic acids or proteins of the pathogens in UTIs have been developed recently [9]–[12]. However, for routine diagnoses in outpatient settings, they are not sufficiently quantitative, easy, or inexpensive. Thus, a rapid and easy method for bacterial detection and bacterial typing has been desired.

In recent years, automated urine particle analyzers have been established as alternative screening methods for urine [13], [14]. The automated urine particle analyzer UF-1000i (Sysmex Corporation, Hyogo, Japan), which incorporates flow cytometry (FCM), has been proposed for bacterial detection at clinically relevant levels [15], [16]. The UF-1000i has a dedicated analytical flow channel named “BACT channel”, which employs specialized reagents and algorithm for bacteria detection and counting. These aspects of UF-1000i realize precise counting of bacteria in urine specimen in a short time. The analyzer has been evaluated in many facilities and has received high commendation for its ability to detect bacteria [17]–[26].

A simple and rapid Gram stain alternative by using UF-1000i would provide important clues for prescribing the appropriate antibiotics for diagnoses of infectious diseases, such as UTIs. Gram-positive and gram-negative refers to how a bacterium reacts to the Gram staining. As is well known, the differences between gram-positive and gram-negative bacteria are based on the outer casing of the bacteria [27]. Gram-positive bacteria have a thick layer of peptidoglycan cell wall, in which the initial Gram stain reagent can be retained. Gram-negative bacteria have a thin layer of peptidoglycan cell wall, which is structurally different from that of gram-positive bacteria, and the walls cannot retain the initial Gram stain reagent. The peptidoglycan cell wall is mechanically and chemically strong in order to protect the bacterial cell, and the cell walls of gram-positive bacteria are more resistant to mechanical or chemical stresses than those of gram-negative bacteria. Therefore, we hypothesized that the differences in the cell wall tolerance to chemicals could be used to distinguish the gram-type of bacteria.

Here, we describe the combined method of simple biochemical pretreatments and bacterial counting with UF-1000i for discriminating between gram-negative and gram-positive bacteria in liquid specimens. This method may be directly applicable to urinalysis.

## Results

### *Escherichia coli* in liquid culture media are easily lysed by the direct addition of an equal volume of NaOH-SDS solution

In order to discriminate between gram-negative and gram-positive bacteria in liquid culture, we hypothesized that differences in cell-wall tolerance to detergent and alkaline pH could be used. For this purpose, we employed the NaOH-SDS solution that was described in a conventional plasmid-extraction method of *E. coli* [28], [29]. This solution can easily lyse *E. coli* when the bacteria are resuspended in a dedicated buffer solution for DNA preparation. However, we did not know whether the solution would lyse bacteria when it was directly added to the growth medium. Furthermore, we were unable to find any literature on this subject.

**Figure 1:**
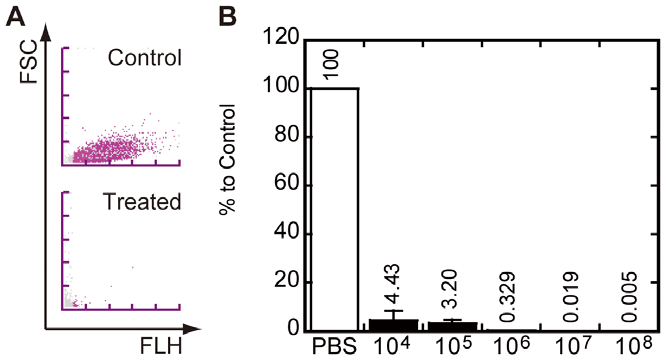
Measurement of the effects of the NaOH-sodium dodecyl sulfate (SDS) lysis solution on cultured *Escherichia coli*. (**A**) Typical BACT scattergrams of the experiment. The *E. coli* culture (2×10^8^ cells/mL) was mixed with an equal volume of phosphate-buffered saline (PBS) (Control) or the NaOH-SDS solution (Treated). Then, the mixture was incubated for 5 min and subjected to UF-1000i. (**B**) The relationship between the range of concentrations of serially diluted *E. coli* culture (1×10^4^ to 10^8^ cells/mL) and the NaOH-SDS solubility. Each symbol represents the average of 3 independent experiments.

In order to confirm if the NaOH-SDS lysis solution was able to lyse *E. coli* when it was directly added to the culture media and to confirm if the UF-1000i could measure the differences that were caused by the addition of the solution, we performed the following experiment. Equal volume of NaOH-SDS solution were added to the mid-log phase cultures of *E. coli*, which were diluted to concentrations that ranged between 1×10^4^ and 1×10^8^ cells/mL with aliquots of fresh medium. After 5 min of incubation at room temperature (RT), the reaction mixtures were measured by the UF-1000i. *E. coli* in the culture medium were completely lysed by the equal volume of the NaOH-SDS solution in this reaction condition (Figure 1 and Table S1). These results clearly showed that the NaOH-SDS solution was able to easily lyse *E. coli* in culture medium when its concentration was at least less than 1×10^8^ cells/mL. In addition, the results showed that the UF-1000i was able to detect the differences before and after treatment with the NaOH-SDS solution.

### The NaOH-SDS solution was a suitable reagent to lyse *E coli* in liquid culture

**Figure 2:**
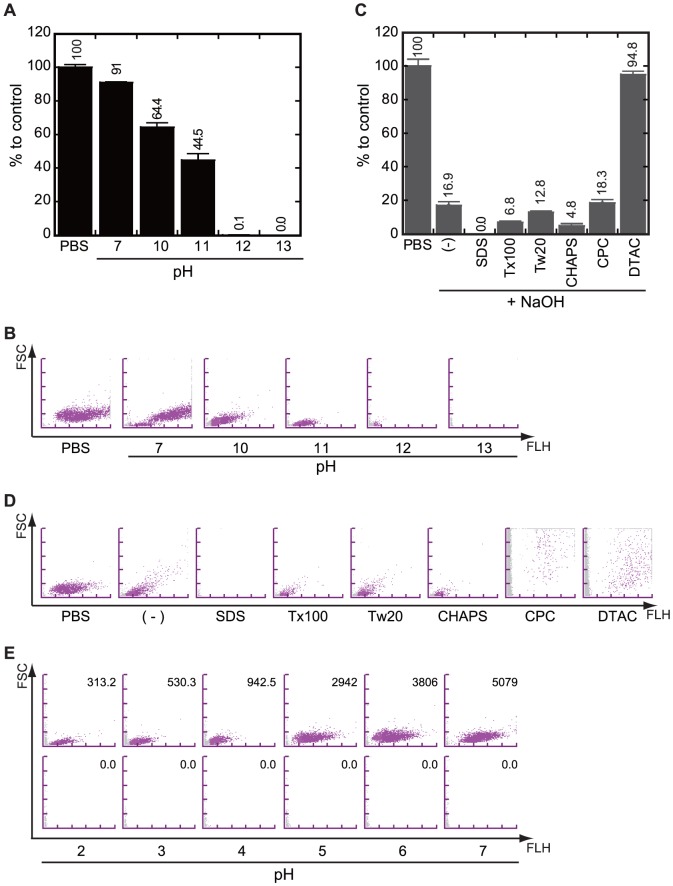
NaOH-SDS solution was a suitable reagent to lyse *E coli* in liquid culture. (**A, B**) The relationship between the solubility of *E coli* and pH of the alkaline SDS lysis solutions with pH between 13 and 10. (**A**) Each bar represents the average of 3 independent experiments, and each error bar represents standard deviation. (**B**) Typical BACT scattergrams of the experiments shown in panel A. The *E coli* culture (4×10^6^ cells/mL) was mixed with an equal volume of phosphate-buffered saline (PBS) as negative control or alkaline-SDS solution. Then, the mixture was incubated for 5 min and subjected to flow cytometry using UF-1000i. (**C, D**) The relationship between the solubility of *E coli* and kinds of detergent in the NaOH-detergent lysis solutions. (**C**) The kinds of detergents are indicated. Each symbol represents the average of 3 independent experiments. (-) indicates no detergent (0.2N NaOH only). (**D**) Typical BACT scattergrams of the experiments shown in panel C. The *E coli* culture (2×10^8^ cells/mL) was mixed with an equal volume of PBS as control or the NaOH-detergent solution. Then, the mixture was incubated for 5 min and subjected to flow cytometry using UF-1000i. Detergents used in the experiment were indicated under the scattergrams. (**E**) Typical BACT scattergrams of the experiments to study the effect of the pH of culture media. The *E coli* culture incubated for 4 h was mixed with an equal volume of PBS or the NaOH-SDS solution. The counted numbers (average of three independent experiments) of *E. coli* are indicated in the scattergrams and the initial pH of the medium are indicated under the scattergrams.

Because the NaOH-SDS solution could be used to lyse *E coli* in liquid culture, we tried to optimize the pH of the lysis solution. Mid-log phase cultures of *E. coli* at concentrations of 4×10^6^ cells/mL were treated with equal volumes of alkaline SDS solutions with pH that ranged between 13 and 10, differing in increments of pH 1, at room temperature (RT) for 5 min. Their count was then compared to that of a negative control diluted with PBS. The results showed that the bacterial count was 40% more than that of the control under pH 11 (Figure 2, panels A and B).

We then tried to optimize the kind of detergent. We used 2 nonionic detergents (polyoxyethylene sorbitan monolaurate [Tween 20] and polyoxyethylene octyl phenyl ether [Triton X-100]), 1 zwitterionic detergent [CHAPS], and 2 cationic detergents (cetylpyridinium chloride monohydrate [CPC] and Dodecyltrimethylammonium Chloride [DTAC]) instead of SDS in the NaOH-SDS lysis solution. Mid-log phase cultures of *E coli* at concentrations of 4×10^6^ cells/mL were treated with equal volumes of NaOH-detergent lysis solution at RT for 5 min, and the bacterial count was compared to that of a negative control. The results indicated that the strongest detergent was SDS (Figure 2, panels C and D).

Next, we tried to check the effect of pH of the culture media because human urine has various pH from 4 to 8. We cultivated *E. coli* in the media at pH 2 to 7 adjusted with hydrochloric acid and counted bacterial numbers after the NaOH-SDS treatment or PBS addition as control. The results clearly showed that the differences of initial pH of the culture media were not affected to lytic activity of the NaOH-SDS solution (Figure 2, panel E).

Thus, we concluded that the original NaOH-SDS lysis solution was the most suitable reagent to lyse *E coli* in liquid culture.

### *Enterococcus faecalis*, a gram-positive bacterium, could not be lysed completely by the NaOH-SDS solution

**Figure 3:**
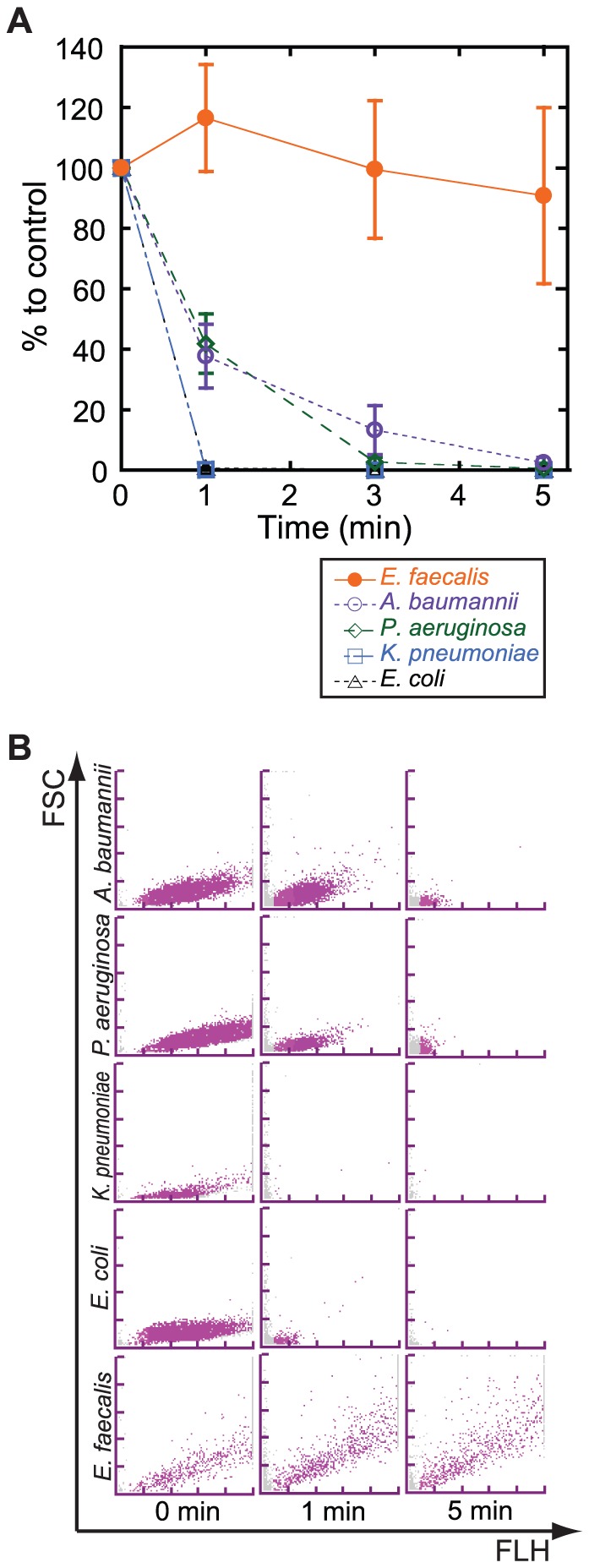
NaOH-SDS treatment lysed gram-negative bacteria within 5 min. (**A**) The mid-log phase culture of 4 gram-negative bacterial strains (*Escherichia coli*, *Klebsiella pneumoniae*, *Pseudomonas aeruginosa*, and *Acinetobacter baumannii*) and *Enterococcus faecalis* (gram-positive bacteria used as control) were treated with the NaOH-SDS solution for the indicated time. The numbers of bacteria that were measured by the UF-1000i were converted into a percentage relative to the control that was treated with an equal volume of PBS. Each symbol represents the average of 3 independent experiments, and the error bars represent the standard deviation of the mean. (**B**) Representative BACT scattergrams of the NaOH-SDS-treated bacterial cultures that were incubated for 1 or 5 min, or control (0 min).

Next, we examined if the NaOH-SDS treatment could be used to differentiate gram-negative and gram-positive bacteria. We chose *E. faecalis* as a representative gram-positive bacterium because it is one of the most frequently detected gram-positive bacterial strains in urine specimens from nosocomial, chronic or recurrent UTI patients [22], [30], [31] as is the case with *S. saprophyticus* [32], [33]. We treated the mid-log phase culture of *E. faecalis* with the NaOH-SDS solution in the same way as that used in the *E. coli* experiment described above. After 5 min of treatment, we confirmed that more than half of the original amounts of *E. faecalis* were detected by the UF-1000i (Figure 3).

### The optimum reaction time of the NaOH-SDS treatment for gram-positive and gram-negative discrimination was 5 min at RT

We then tried to optimize the reaction time of the NaOH-SDS treatment. As described above, *E. coli* was easily lysed in 5 min at RT by the treatment, whereas *E. faecalis* was not completely lysed by the same treatment. We supposed that the gram-negative bacteria would generally be easily lysed and that gram-positive bacteria would not. Thus, we divided this experiment into 2 parts.

First, we tried to determine the minimum reaction time required to lyse gram-negative bacterium by using 4 strains of gram-negative bacteria (*E. coli, Klebsiella pneumoniae, Pseudomonas aeruginosa, and Acinetobacter baumannii*). Mid-log phase cultures of the bacteria were treated with NaOH-SDS solution for 1, 3, and 5 min at RT, and their numbers were calculated by the UF-1000i. Then, the numbers were compared with that of a negative control, which was made by diluting the original culture with the addition of an equal volume of phosphate-buffered saline (PBS). The results showed that all 4 bacterial numbers were reduced to less than 30% or 10% compared to the negative control by 3 or 5 min of reaction, respectively (Figure 3).

**Figure 4:**
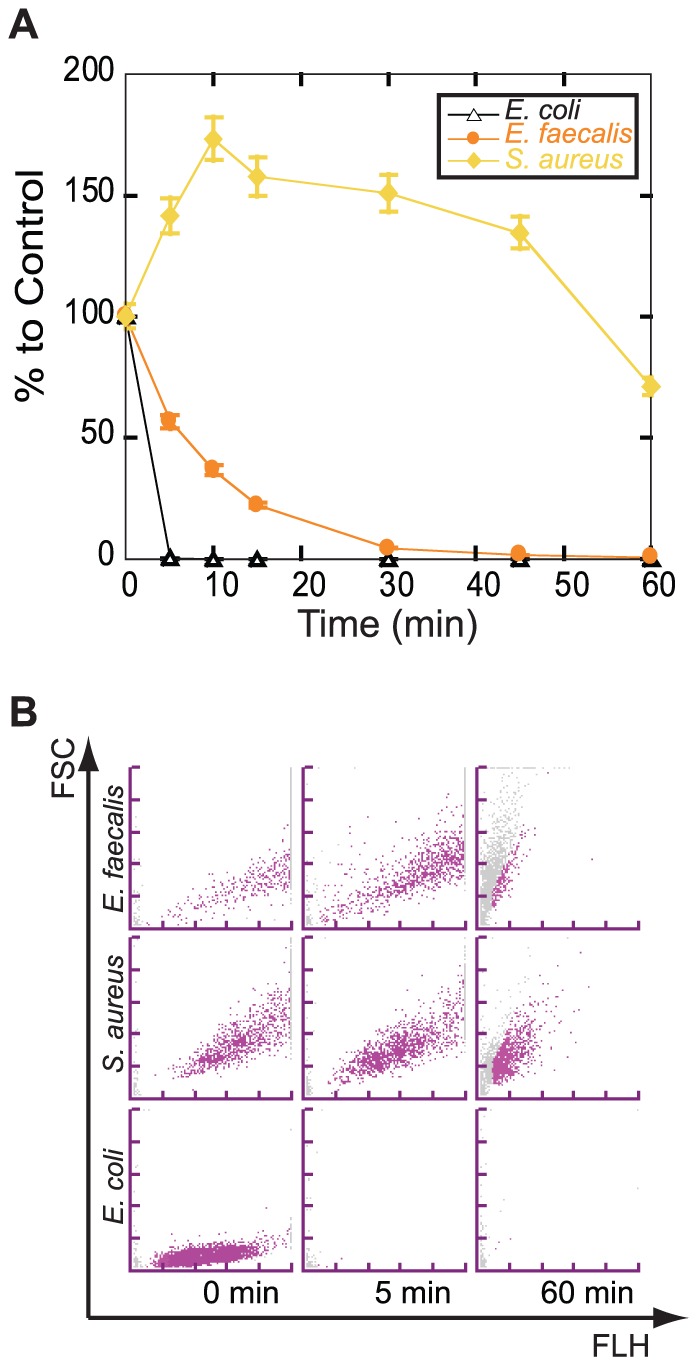
NaOH-SDS treatment did not lyse gram-positive bacteria in 5 min. (**A**) The mid-log phase culture of 2 gram-positive bacterial strains (*Staphylococcus aureus and Enterococcus faecalis*) and *Escherichia coli* (gram-negative bacteria used as control) were treated with the NaOH-SDS solution for the indicated time. The numbers of bacteria that were measured by the UF-1000i were converted into percentages that were relative to the control that was treated with an equal volume of PBS. Each symbol represents the average of 3 independent experiments, and the error bars represent the standard error of the mean. (**B**) Representative BACT scattergrams of NaOH-SDS-treated bacterial culture incubated for 5 or 60 min or the control.

Second, we tried to determine the maximum reaction time for gram-positive bacteria by using 3 strains of gram-positive bacteria (*E. faecalis*, *Staphylococcus aureus*, and *Lactobacillus acidophilus*). Mid-log phase cultures of these bacteria were treated with the NaOH-SDS solution for 5, 10, 15, 30, 45, and 60 min at RT, and their numbers were calculated by UF-1000i. Then, their numbers were compared to that of a negative control diluted with PBS. The results showed that all 3 bacterial numbers were retained more than 50% and 20% compared to that of the control by 5 and 10 min of reaction, respectively (Figure 4).

These results showed that the optimum reaction time for Gram discrimination was 5 min at RT and indicated that the most sensitive gram-positive bacterium was *E. faecalis*. Moreover, although all of these gram-positive bacteria were lysed after 60 min of reaction, they showed a transient increases in their numbers just after the addition of the NaOH-SDS solution (Figure 4).

### Clusters of gram-positive bacteria were divided by the NaOH-SDS treatment

**Figure 5:**
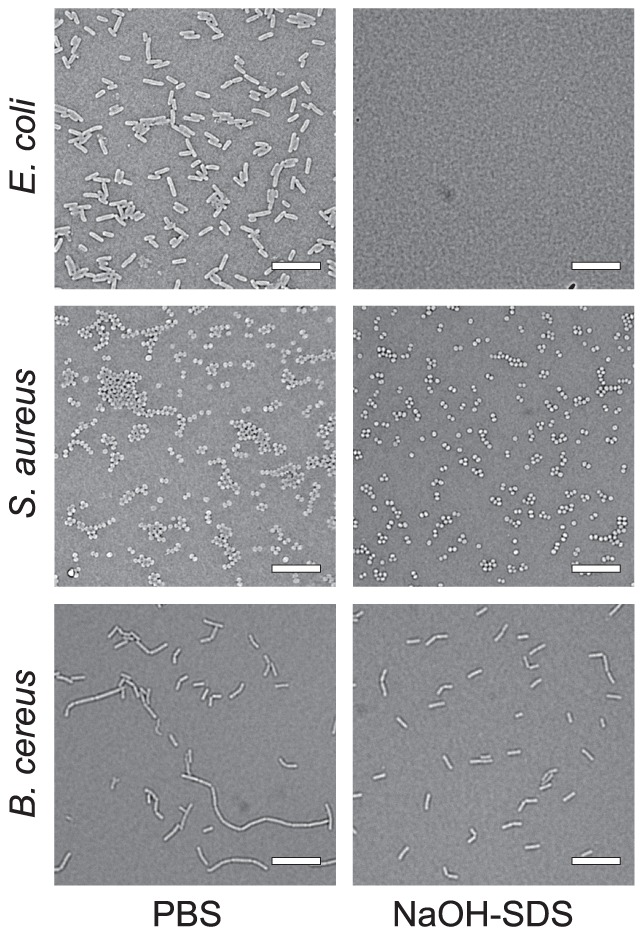
Microscopic observations of the bacterial cells before and after treatment with the NaOH-SDS solution. The upper panels show *Escherichia coli*, the middle ones show *Staphylococcus aureus*, and the lower ones show *Bacillus cereus*. The left panels show the negative control, and the right ones show the samples that were incubated with NaOH-SDS for 5 min. Bar = 10 µm.

In order to determine the reason for the transient increases in the numbers of gram-positive bacteria after the NaOH-SDS treatment, we performed microscopic observations using the gram-positive bacteria, *S. aureus*, and the gram-negative bacteria, *E. coli*. Before the treatment, *S. aureus* formed large clusters that were composed of many bacterial cells, but, in contrast, almost all *E. coli* existed as individual cells or as 2-cell conbinations (Figure 5, left panels). After the NaOH-SDS treatment, cell clusters of gram positive bacteria, *S. aureus* and *B. cereus*, were disrupted into smaller groups, whereas *E. coli* cells completely disappeared (Figure 5, right panels). These results indicated that the transient increase of the gram-positive bacteria by the NaOH-SDS treatment was likely due to the disruption of bacterial clusters.

### Gram-positive bacteria and gram-negative bacteria were discriminated by their differing tolerance to the NaOH-SDS treatment

**Figure 6:**
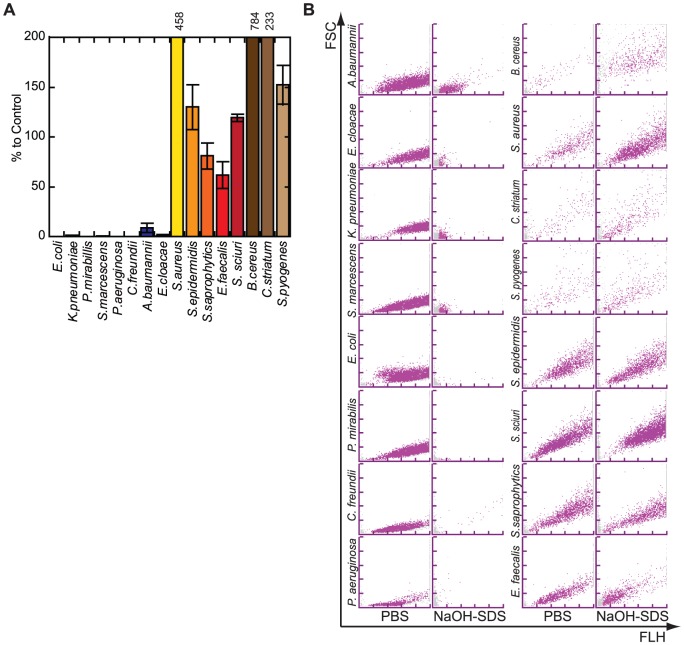
Reactivity to the NaOH-SDS solution reflects the Gram stainability of bacteria. (A) The mid-log phase culture of 8 gram-positive bacteria (*Bacillus cereus*, *Staphylococcus aureus*, *Corynebacterium striatum*, *Streptococcus pyogenes*, *Staphylococcus epidermidis*, *Lactobacillus acidophilus*, *Staphylococcus saprophyticus*, and *Enterococcus faecalis*) and 8 gram-negative bacteria (*Acinetobacter baumannii*, *Enterobacter cloacae*, *Klebsiella pneumoniae*, *Serratia marcescens*, *Escherichia coli*, *Pseudomonas aeruginosa*, *Citrobacter freundii*, and *Proteus mirabilis*) were treated with the NaOH-SDS solution for 5 min. The numbers of bacteria that were measured by the UF-1000i were converted into percentages that were relative to the control that was treated with an equal volume of PBS. Note that the vertical axis was up to 200%. Each column represents the average of 3 independent experiments and each error bar shows the standard deviation. Numbers indicated above columns show the average of 3 independent experiments. (**B**) Representative BACT scattergrams of NaOH-SDS-treated bacterial culture incubated for 5 min or controls.

In order to evaluate the generalizability of this method, we treated 8 clinically important gram-positive bacterial strains and 8 important gram-negative bacterial strains in the same way. The results clearly showed that all of the gram-positive bacteria were more resistant to the NaOH-SDS treatment than the gram-negative bacteria, and the border of the 2 bacterial subtypes was around 25% of the control (Figure 6).

**Figure 7:**
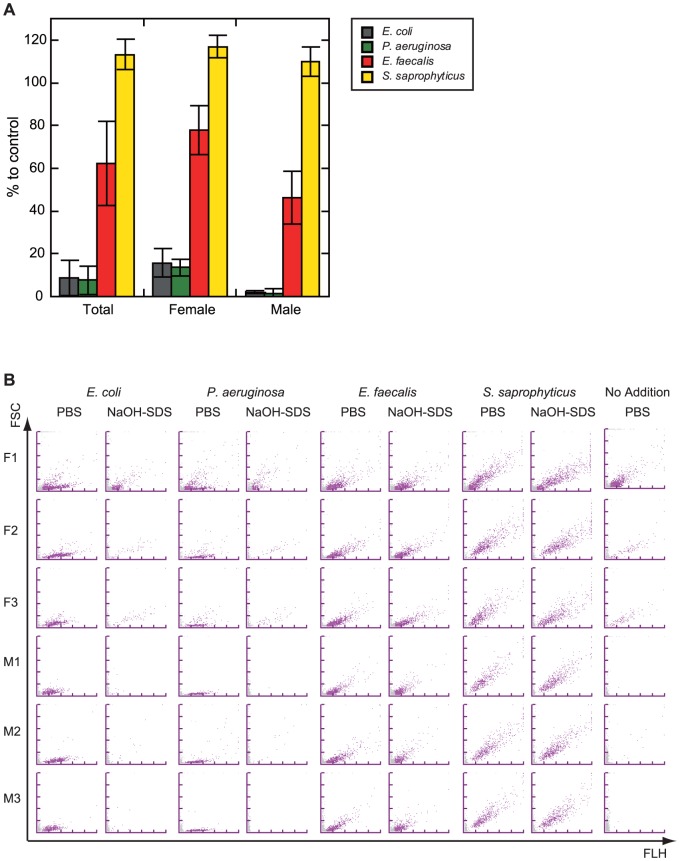
Bacteria cultured in urine show the same reactivity to the NaOH-SDS solution. (A) The mid-log phase culture of 2 gram-positive bacteria (*Staphylococcus saprophyticus*, and *Enterococcus faecalis*) and 2 gram-negative bacteria (*Escherichia coli* and *Pseudomonas aeruginosa*) cultured in voluntary urine from 3 male and 3 female human volunteers were treated with the NaOH-SDS solution for 5 min. The numbers of bacteria that were measured by the UF-1000i were converted into percentages that were relative to the control that was treated with an equal volume of PBS. Each column represents the average of 6 (Total) or 3 (Female and Male) independent urine cultures and each error bar shows the standard deviation. (**B**) BACT scattergrams of NaOH-SDS-treated bacterial culture incubated for 5 min or controls.

Finally, we tried to estimate the potential matrix-effect of urine on our method. For this purpose, we used bacterial culture incubated in voluntary urine from healthy volunteers instead of culture medium (Figure 7). The results also clearly indicated that the NaOH-SDS treatment selectively lyse gram negative bacteria. Note that the urine, especially from female volunteers, contained significant numbers of contaminated bacteria. After incubation, the contaminated bacteria proliferated in significant numbers and somewhat affected the results (Figure 7 panel A and panel B right row). Note that the voluntary urine from women volunteers has much contamination other than bacteria (Figure S3). In spite of the contamination, we found that there is a clear border between gram negative and gram positive bacteria in the ratio to control (Figure 7 panel A).

Thus, we concluded that this method, the NaOH-SDS treatment followed by UF-1000i measurement, was a rapid, simple, and effective technique for predicting the Gram stainability of bacteria in liquid culture.

## Discussion

In this paper, we described a rapid and simple method for discriminating gram-positive and gram-negative bacteria in liquid media. This method was based on 2 components. The first was the use of the NaOH-SDS lysis solution of the plasmid extraction method for Gram discrimination. The NaOH-SDS lysis solution was developed for the plasmid extraction method of *E. coli* more than 30 years ago [28], [34]. The method, which is also called the alkaline method, is by far the most popular method because of its simplicity, relatively low cost, and reproducibility. However, it is well known among molecular biologists that have experience studying gram-positive bacteria that the NaOH-SDS lysis solution of the original plasmid extraction method for *E. coli* does not lyse gram-positive bacteria effectively [35], [36]. The second was the use of the automated urine particle analyzer UF-1000i as a flow cytometer for bacterial cell counting. The UF-1000i can count the numbers of bacteria in a urine specimen in 1 min with its separate flow channel in which the nucleic acids of the bacteria are stained with a specific fluorescent dye and discriminated from cell debris [13]. The results were shown in the main window of its analytical software and the operator can get the numbers of bacteria and the pattern of the bacterial counting channel at a glance (Figure S3).

Because our aim was to invent a novel method that can be applied to routine clinical diagnosis, we wanted to make the method as simple as possible. In the first part of the results, we described that the NaOH-SDS solution was able to easily lyse *E. coli* when an equal volume of the solution was added directly into the liquid culture (Figure 1). In the conventional plasmid extraction method of *E. coli*, the bacteria in the liquid culture are collected by centrifugation and resuspended in a dedicated solution containing Tris buffer (pH 8.0), sugar (glucose or sucrose), and chelator (EDTA or CDTA) to adjust the pH of the reaction solution before the addition of the NaOH-SDS lysis solution [28], [29], [34]. In addition, we described that the number of bacteria before and after treatment with the NaOH-SDS solution can be easily counted without any pH adjustment by the automated urine flow cytometer UF-1000i (Figure 1). The dilution reagent used in BACT channel is acidic (pH 2.5) for hemolysis and specific staining of bacteria. On the other hand, our method recruited 200 mM NaOH. So we were afraid that the basic pH of our NaOH-SDS reagent might affect the analysis by the BACT channel. But, as a result, the analysis was done with no problem. From the viewpoint of developing a simple method, these 2 successes were small but important achievements. In addition, we re-confirmed that the NaOH-SDS solution was a suitable reagent to lyse *E coli* in liquid culture (Figure 2) [28], [29].

In the second part, we confirmed that *E. faecalis*, which is the most frequently detected gram-positive bacteria in urine specimens from nosocomial, chronic or recurrent UTI patients [22], [22], [30,31], was not completely lysed with the same procedure of the NaOH-SDS treatment (Figure 3). In addition, the results showed that the UF-1000i had the ability to quantify the differences in reactivity to the NaOH-SDS solution between *E. coli* and *E. faecalis* (Figure 3). Thus, we concluded that this combination method might have the ability to differentiate gram-negative and gram-positive bacteria in liquid culture.

We then investigated the optimum reaction time of the NaOH-SDS pretreatment required to discriminate gram-positive and gram-negative bacteria. First, we tried to determine the minimum reaction time for gram-negative bacteria and found that 5 min was enough reaction time to lyse the gram-negative bacteria that we examined (Figure 3). The most resistant strain of the 4 gram-negative bacterial strains was *A. baumannii*. This result seemed to be consistent with the well-known facts that *A. baumannii* have gram-variable properties and are sometimes stained by Crystal violet. Second, we tried to find the maximum reaction time for gram-positive bacteria and found that 5 min was a suitable reaction time for gram-positive bacteria because most of the *E. faecalis* was lysed when the reaction time was extended for more than 10 min (Figure 4). Thus, we concluded that the optimum incubation time at RT was 5 min.

Through the experiments described above, we noticed that the numbers of all 3 gram-positive bacterial strains were transiently increased after NaOH-SDS treatment. In contrast, we did not observe any increases in the numbers when we treated the gram-negative bacteria. In order to determine the reason for the transient increase of gram-positive bacteria, we performed microscopic observations of both *E. coli* and *S. aureus* and found that the clusters of the gram-positive bacteria, *S. aureus*, were divided into smaller groups after treatment with the NaOH-SDS solution, and the gram-negative bacteria, *E. coli*, disappeared completely with the treatment (Figure 5). These results suggest that the UF-1000i flow cytometer detects each cluster of gram-positive bacteria as an individual particle and not as a multicellular cluster. However, considering semiquantitative bacterial culture on agarose plates, such bacterial clusters may not be broken through the processes of cultivation because the cells in the clusters connect directly and tightly through their cell walls [37]. These hypotheses are consistent with previous results that showed that linearity of the bacterial measurement was observed in both *E. coli* and *S. aureus*. We tried to count the colony number of the NaOH-SDS treated bacteria. Although we neutralize the samples after 5 min reaction of NaOH-SDS by various concentrations of hydrochloric acid or acetic acid and plated them onto HIF-agar plate, no colonies were appeared from the NaOH-SDS treated samples. Maybe the damage of the treatment is enough to kill the bacteria, or the combination of the treatment and plating on the agar plate is harmful for the bacteria. Thus, we concluded that the transient increase in the numbers of gram-positive bacteria after NaOH-SDS treatment was mainly due to disruption of the cell clusters.

On the courses of these experiments, we noticed that some scattergrams of the same bacterial strain in the same experimental condition showed somewhat different pattern (for example, control of Figure 2B and 2D). We then found that staining intensity of bacteria by the reagents used in BACT channel is changed by the growing state of bacteria (Figure S1).

Then we tried to clarify whether this method could be done by a general purpose flow cytometer, FACSCalibur (BD Biosciences). Although we could reproduce the BACT scattergram with control bacteria, we could not reproduce the BACT scattergram by using NaOH-SDS treated bacteria (Figure S2). In the automatic urine particle analyzer UF-1000i, the reaction condition is completely controlled among the samples and we could not reproduce the condition manually. Moreover, the hardware of UF-1000i is specially designed for bacterial detection; i.e., an independent flow channel for bacteria, specially regulated flow rate, and the oval shape of laser-beam for small particles, and so on.

Finally, we tried to evaluate the generalizability of the method by using 8 gram-positive and 8 gram-negative strains cultured in heart infusion medium (Figure 6), or by using 4 gram-negative and 4 gram-negative strains incubated in voluntary urine from 6 healthy volunteers (Figure 7). The results indicated that the two bacterial subtypes were clearly discriminated by their reactivity to NaOH-SDS treatment estimated by UF-1000i. Bacteria cultured not only in culture broth but also in voluntary urine from healthy volunteers (Figure 6 and Figure 7), even in the urine with bacterial contamination, white blood cells or any other urinary precipitations (Figure 7 and Figure S3), showed clear Gram-subtype difference. Although these results were not enough to establish the generalizability of the method, they suggested the possibility that the method can be directly applicable to liquid clinical specimens, such as urine or other body fluids. Moreover, because the UF-1000i counts the numbers of bacteria in urine within a minute with no pretreatment, medical staff without any special skills or experiences may be able to predict the Gram subtype of bacteria in fluid specimens in less than 10 min with this combination method of the NaOH-SDS treatment and UF-1000i measurement.

This rapid and simple method may be easily applied to obtain additional information from bacteriuria. It may be useful for determining the treatment course of patients with UTIs, especially for patients who do not have a prior history of UTIs. Because the single analysis cost of UF-1000i is as the same level as the cost of gram staining in our estimation, and because the single analysis cost of NaOH-SDS reagent is much cheaper, our gram discrimination method must be cost-effective. Further evaluations of this method using clinical specimens are challenges of the near future.

## Materials and Methods

### Bacterial strains and media

16 reference bacterial strains, *Escherichia coli* ATCC25922, *Klebsiella pneumoniae* ATCC13883, *Proteus mirabilis* ATCC25933, *Serratia marcescens* ATCC13880, *Pseudomonas aeruginosa* ATCC 27853, *Citrobacter freundii* ATCC8090, *Acinetobacter baumannii* ATCC19606, *Enterobacter cloacae* ATCC23355, *Staphylococcus aureus* ATCC29213, *Staphylococcus epidermidis* ATCC12228, *Staphylococcus saprophyticus* ATCC15305, *Enterococcus faecalis* ATCC29212, *Staphylococcus sciuri* JCM2425, *Bacillus cereus* ATCC14579, *Corynebacterium striatum* ATCC BAA1293, and *Streptococcus pyogenes* ATCC BAA946 were used. All bacterial stocks were cultured on Heart Infusion agar plates (51047, Nissui Pharmaceutical Co, Ltd, Tokyo, Japan). The colonies were picked up and preincubated in Heart Infusion broth (05505, Nissui Pharmaceutical Co, Ltd) for more than 16 h in order to prepare the preculture. The aliquots of preculture were then diluted in fresh Heart Infusion broth and cultured for about 4 h for the experiments of mid-log phase culture. In addition, we prepared modified Heart Infusion broth that adjusted their pH at 2, 3, 4, 5 and 6 with hydrochloric acid. These culture media were sterilized by filtration.

### Reagents

NaOH, SDS, polyoxyethylene sorbitan monolaurate [Tween 20], polyoxyethylene octyl phenyl ether [Triton X-100], 3-[(3-Cholamidopropyl)dimethylammonio]-1-propanesulfonate [CHAPS], and Agarose-LE were purchased from Nacalai Tesque, Inc, Kyoto, Japan. PBS (16219321), cetylpyridinium chloride monohydrate [CPC], and Dodecyltrimethylammonium Chloride [DTAC] was purchased from Wako Pure Chemical Industries, Ltd, Osaka, Japan. Water was made by direct-Q (Millipore, Billerica, MA, USA). The NaOH-SDS solution (0.2 N NaOH, 1% SDS) [28] was prepared daily from both a 10 N NaOH and a 10% SDS stock solution. Other buffers or detergents were also used in 0.2 N or 1%, respectively. In addition, we used CAPS-NaOH buffer for pH 10 and 11, and NaOH-Na_2_HPO_4_ buffer for pH 12 and pH 13.

### Urine culture

Voluntary urine from 6 healthy volunteers (3 female and 3 male; with informed consent) was collected in urine collection receptacle. The urine was mixed well and subdivided into 6 culture tubes. The aliquots of preculture of 4 bacterial strains (*E. coli*, *P. aeruginosa*, *E. faecalis* and *S. saprophyticus*) in Heart Infusion broth were diluted in urine and cultured for about 4 h for the experiments of mid-log phase culture. This study was reviewed by the Sysmex Ethics Committee. All volunteers gave informed consent according to the study protocol.

### NaOH-SDS treatment

A 0.5-mL aliquot of mid log-phase bacterial culture was transferred into a 1.5-mL tube. Then, 0.5 mL of the NaOH-SDS solution, or PBS for negative control, was added. The tube was mixed upside down 6 times and incubated at RT (20°C–25°C). In the standard procedure, the mixture was incubated for 5 min. In the time-course experiment, the incubation time was varied from 1 to 60 min as described in the figure legends. Immediately after the incubation, each sample was examined by the UF-1000i system as described below.

### Flow cytometry

The automated urine particle analyzer UF-1000i (Sysmex Corporation, Hyogo, Japan) was used as a flow cytometer. All reagents used in the UF-1000i were from Sysmex Corporation. Testing with the UF-1000i system was done according to the instructions provided by the supplier. The BACT channel of the UF-1000i was used for bacterial counting. In the scattergram of the BACT channel (the BACT scattergram), the vertical axis shows the intensities of forward scatter, and the horizontal axis shows the intensities of the fluorescent stain that represents the amounts of nucleic acid.

### Microscopy

*E. coli* or *S. aureus* samples, which were treated with the NaOH-SDS solution or diluted with PBS (control), were observed using agarose gel-coated slide glass as described previously [38], with slight modifications. Briefly, the slide glass (Matsunami Glass Ind, Ltd, Osaka, Japan) was covered with 2 mL of 2% (w/v) agarose gel, dried at RT for more than 1 day, and stored at RT. About 0.01 mL of bacterial culture was dropped on the agarose-coated slide glass and covered with a coverslip (Matsunami Glass Ind, Ltd). After a few min of incubation, the slide was observed under a microscope (BX-60 microscope with UPLAPO100XO lens, Olympus Corporation, Tokyo, Japan). The images were acquired with a DP25 digital CCD camera (Olympus Corporation).

## Supporting Information

Figure S1 **The relationship between growing state of bacteria and BACT scattergram pattern.** (A, B) Growth curve of the four gram-negative (A) or four gram-positive (B) bacteria. (C, D) BACT scattergrams of each time points.
(EPS)

Figure S2 **BACT scattergrams reproduced with general purpose flow cytometer.** Control (left panels) or NaOH-SDS treated (right panels) bacterial cultures (Top: *E. coli*, middle: *S. saprophyticus*, bottom: *E. faecalis*) were analyzed by using FACSCalibur.
(EPS)

Figure S3 **Main window from the automated urine particle analyzer UF-1000i.** The main windows of UF-1000i control software of (A) control urine from a female volunteer stored at 4°C, (B) urine incubated at 37°C for a few hours with *Enterococcus faecalis* diluted with PBS or (C) urine with *E faecalis* treated with NaOH-SDS. Red rectangles in panel A indicate the BACT scattergram and the number of bacteria counted by BACT channel and black arrowhead (B and C) indicates the region of white blood cells which lysed with NaOH-SDS treatment.
(EPS)

Table S1 **Numbers in** **Figure 1** **panel B.**
(TIF)
