# Supplementary figures and images for: Rapid Discrimination of Gram-Positive and Gram-Negative Bacteria in Liquid Samples by Using NaOH-Sodium Dodecyl Sulfate Solution and Flow Cytometry
Source: PLoS One. 2012 Oct 15;7(10):e47093. doi: 10.1371/journal.pone.0047093 (PMC3471971; doi:10.1371/journal.pone.0047093)

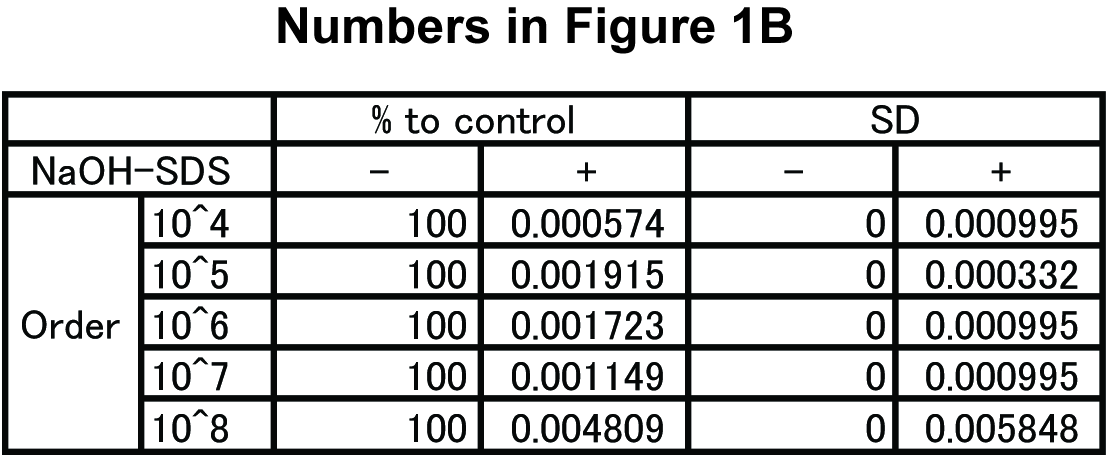

Supplement: Table S1 — Numbers in Figure 1 panel B. (TIF) [file pone.0047093.s004.tif]
